# First Bites—Why, When, and What Solid Foods to Feed Infants

**DOI:** 10.3389/fped.2021.654171

**Published:** 2021-03-26

**Authors:** Stephen M. Borowitz

**Affiliations:** Division of Pediatric Gastroenterology, Hepatology and Nutrition, University of Virginia, Charlottesville, VA, United States

**Keywords:** infant feeding, weaning, infant nutrition, complementary feeding, taste, solid foods, food preferences

## Abstract

Humans are the only mammals who feed our young special complementary foods before weaning and we are the only primates that wean our young before they can forage independently. There appears to be a sensitive period in the first several months of life when infants readily accept a wide variety of tastes and this period overlaps with a critical window for oral tolerance. As a result, infants should be exposed to a wide variety of flavors while mother is pregnant, while mother is nursing and beginning at an early age. There also appears to be a sensitive period between 4 and 9 months when infants are most receptive to different food textures. There remains debate about when it is best to begin introducing solid foods into an infant's diet however, the available evidence suggests that provided the water and food supply are free of contamination, and the infant is provided adequate nutrition, there are no clear contraindications to feeding infants complementary foods at any age. There is emerging evidence that introduction of solid foods into an infant's diet by 4 months may increase their willingness to eat a variety of fruits and vegetables later in life, decrease their risk of having feeding problems later in life, and decrease their risk of developing food allergies, and the early introduction of solid foods into an infant's diet does not appear to increase their risk of obesity later in childhood.

## Introduction

Humans are the only mammals that feed our young complementary foods before weaning and the only primates that wean our offspring before they can forage independently (1). The human anomaly of early weaning is likely due to our slow post-natal growth and maturation which in turn are likely due to our brain size. The human brain is more than three times larger than our closest relative the chimpanzee. In human adults, the brain accounts for 2% of body mass but consumes 25% of resting energy expenditure. An average adult needs 600 kilocalories per day to feed his or her brain (2). In other primates, the brain consumes <10% of resting energy expenditure (3).

In non-human primates, time spent feeding increases with body mass; the bigger the animal, the more time spent feeding. On a raw diet similar to what non-human primates eat, humans would need to feed 12 h every day to meet our energy needs however, the actual value is only an hour (4). We solved this paradox with cooking; cooking enables us to eat a diet much lower in fiber than any other ape. The move from raw food to cooked food was associated with a significant reduction in gut size as compared to our ancestors and allowed us to divert more energy to brain growth (5) and increased the time available for social and other cognitively demanding activities which in turn imposed positive pressure for increasing brain size (6).

Humans have the longest period of juvenile dependency of all primates, and the shortest period of lactation. If humans followed the great ape pattern of supplementing our offspring via lactation and not conceiving again until juveniles were nutritionally independent, our interbirth interval would be between 10 and 15 years. Early weaning allows human mothers to resume reproduction much sooner than expected. Moreover, human mothers are unique in that they simultaneously care for multiple dependent offspring at different stages of maturity (7). Cooking and making food more digestible allows children to be weaned onto adult foods much more quickly and allows mothers to stop nursing sooner; all human societies prepare some form of weaning food (7, 8).

## When Do We Start Feeding Infants Solid Foods?

As a result of feeding our young complementary foods, there does not appear to be a specific age for weaning in humans, and weaning is variable across cultures. In pre-industrial populations, complementary foods are usually started between 4 and 6 months and breast-feeding has completely ceased by 30 months however, in some cultures solids are started substantially earlier (1). In general, the more active women are in subsistence activities, the earlier supplementary foods are started however, there is variation that seems the result of the work women are expected to do (9).

Since 2002, the WHO has recommended infants be exclusively breast fed for the first 6 months of life to optimize their growth, development and health (10). The American Academy of Pediatrics, the American College of Obstetrics and Gynecology and the American Academy of Family Physicians all recommend solid foods be introduced at approximately six months of age (11–13) whereas, the European Society for Pediatric Gastroenterology, Hepatology and Nutrition (ESPGHAN) recommends “complementary foods should not be introduced before 4 months but should not be delayed beyond 6 months” (14). Despite these recommendations, in the U.S., 40% of mothers introduce solids before 4 months of age, and in those who start solids before 4 months, the average age is 12 weeks, and 9% of mothers start solids before 4 weeks of age (15). In Australia, 50% of mothers have fed their babies solids by 4 months of age, and 90% of mothers feed solids before 6 months (16). Comparable patterns are reported in the U.K. and Italy (17). In all these studies, the most commonly cited reasons for starting solids early are the baby was hungry all the time, the baby seemed interested in eating solids and/or starting solids would help the baby sleep (15–17). There are studies that confirm that the early introduction of solid foods is associated with longer infant sleep duration and less frequent waking at night, (18, 19). It is worth noting that most of the studies examining when infants commence eating solid foods describe feeding practices before 2010, and the increased emphasis on the benefits of exclusive breast feeding over the past decade may have changed mothers' behaviors.

## What Solid Foods Should We Feed Infants?

Fetuses and newborn infants appear to have an innate preference for sweet and an innate distaste for bitter and other taste preferences appear to be learned through exposure and experience (20, 21). In a study at the Pennsylvania Hospital, newborns were offered water or sucrose solution and allowed to drink for 5 minutes. The infants took nearly three times as much of the sucrose solutions as water. At 6 months, the investigators managed to recall 131 of 151 infants. At the time, many parents fed infants water sweetened with sucrose, honey or Karo syrup as a supplement. The infants who had been fed sweetened water as newborns drank significantly more of the sucrose solution than infants who had not been getting sweetened water as newborns (22).

*In utero* events and exposures appear to influence taste and flavor preferences (23). What mothers eat during pregnancy influences the flavors the fetus is exposed to in the amniotic sac (24). In one study, prior to undergoing amniocentesis, women were randomized to eat garlic capsules or placebo. The smell of garlic was easily detected in the fluid of the women who had eaten garlic but not in those who had not (25). Fetal swallowing frequency increases when they are exposed to sweet solutions in the amniotic fluid and swallowing frequency decreases when they are exposed to bitter solutions, and similar behaviors are observed within hours of birth (26). Newborn infants orient and begin mouthing when exposed to anise or garlic if their mothers had consumed anise or garlic during pregnancy (23, 27).

These prenatal exposures may have long lasting effects (28). At 35 weeks gestation, Irish mothers were randomized to eat four meals a week containing fresh garlic or to not eat any fresh garlic. Eight years later, their children were given a test meal containing two portions of potato gratin, one which contained garlic and one which did not. Remarkably, the children whose mothers ate garlic during the last month of pregnancy ate twice as much of the garlic containing potatoes as did the control group (29). Analogously, what mother eats and drinks while she is nursing influences the flavor of her breast milk (30) and these taste experiences appear to influence her infant's acceptance of new flavors and what flavors they will accept and like later in life (31).

There appears to be a sensitive period during the first several months of life when infants are most receptive to a wide diversity of flavors, and what they taste during this sensitive period can influence their taste preferences later in life (32). Most infants <4 months of age readily drink formulas that contain hydrolyzed casein which are extremely bitter, however beyond 6 months, infants who have never been exposed to these formulas refuse to drink them (33). Moreover, infants fed hydrolysate formulas in the first several months of life are more willing to eat savory, sour or bitter foods than are infants fed standard milk-based formulas and as compared to children who were never fed a hydrolysate formula, 5 year old children who were fed a hydrolysate formula during infancy more readily eat foods with sour or bitter tastes or aromas (34).

Repetition of taste exposures seems to matter as well. In one study, mothers of seven month old infants were asked to identify a vegetable their infant disliked and were instructed to offer that vegetable on alternate days for 16 days, and to offer a well-liked vegetable, typically something sweet like carrots, sweet potatoes or squash, on the other days. On the first day, infants ate substantially less of the disliked vegetable than the well-liked vegetable however by the eighth repetition, the intake of the liked and unliked vegetables were identical (35). A number of studies have demonstrated similar findings with anywhere between five and ten repetitions (36, 37).

In another study 5 month old infants were randomized to three patterns of introduction of complementary foods. A third were randomized to a “no variety group” and got carrot puree every day for 12 days, a third were randomized to a “low variety group” and got puree of carrot, artichoke, green bean, and pumpkin each for 3 days, and the final third were randomized to a “high variety group” and got carrot puree the first day, followed by artichoke, green beans and pumpkin and back to carrot, etc. When the children were six, they were offered a test meal and the children who had experienced a wider variety of vegetables at weaning ate far more new vegetables than those who had experienced little or no variety at weaning (38).

There also appears to be a sensitive period when infants are particularly receptive to different food textures. Infants and young children typically prefer smooth foods over foods with lumps or chunks however, most fruits and vegetables have complex textures which require the infant to use their tongues to move the food around their mouths in preparation for swallow. Some authors suggest infants are not ready to eat solids before 6 months of age when they have developed a phasic bite and release, have good head control, have good trunk stability and are sitting with minimal support, and are bringing their hands and other objects to their mouth however, the ability of infants to use their tongues and move food around their mouth appears to be more dependent upon their experiences with textured food than on any particular age or developmental stage (39). When infants are exposed to a variety of textures, they are more willing to eat and enjoy chopped or chunky foods at 12 months (40), are more likely to eat a variety of fruits and vegetables at 7 years, and are less likely to suffer from feeding problems during childhood (41). In contrast, children who are not introduced to solids until after a year are more likely to develop oral defensiveness and refuse more highly textured foods (42).

## Potential Risks of Early Introduction of Solids

### Allergies

It has long been proposed that early introduction of solids increases the risk of allergies later in life (43) however, evidence is accumulating that early introduction of solids may decrease the risk of food allergies (44). In the LEAP study, 640 infants with eczema, egg allergy or both were randomized to avoid peanuts or to consume a minimum amount of peanut containing foods. At 5 years of age, the proportion of children who had peanut allergy as assessed by oral food challenge was substantially lower in the peanut consumption group (45). In a follow-up study a year later, the findings were unchanged (46). In the EAT study 1,303 exclusively breastfed infants were introduced to six allergenic foods (peanut, cooked egg, cow's milk, sesame, whitefish and wheat) at 4 months, or were exclusively breast fed through 6 months. At 36 months, 2.4% of the early exposure group was allergic to one or more food as compared to 7.3% of the group who were introduced to solids after 6 months, and the early exposure group had significantly lower rates of peanut, egg, and milk allergy (47).

These findings suggest there is a window for oral tolerance during early infancy that begins to close somewhere around 6 months of age. This comports with animal models suggesting tolerance is dependent upon exposure to exogenous proteins coupled with development of a healthy intestinal microbiome during a critical early window in life (48).

### Obesity

There is little evidence early introduction into an infant's diet increases their subsequent risk of obesity (49). In one trial, 165 formula fed infants were randomized to be fed solid foods beginning at 3 months as compared to 6 months of age. Weights were identical at 3, 6, and 12 months of age (50). In a Scandinavian study, 100 breast-fed infants were randomized to receive complementary foods beginning at 4 months or to continue to exclusively breast feed through 6 months. Weights at 9, 12, and 36 months were identical in the two groups (51). In the EAT study described above, exclusively breastfed infants in the UK were introduced to six allergenic foods at 4 months, or were exclusively breast fed through 6 months and the weights of the two groups were identical at 3, 12, and 36 months (47). The majority of studies show no association between introduction of solids at 4 months and an increased risk of obesity later in childhood. There are several retrospective observational studies suggesting there may be an increased risk of obesity when solids are introduced before 4 months however, it is important to consider the possibility of reverse causality as rapid weight gain and size at 6 months are independent risk factors for future obesity (52). Moreover, it is important to remember that the most common reason parents cite for starting solids early is because they perceive the baby as being hungry much of the time, whether this is right or wrong (15–17).

### The Intestinal Microbiome

During exclusive breast or formula feeding, the infant's intestinal microbiome contains a pre-ponderance of bifidobacteria and enterobacteriacae with smaller numbers of streptocci, lachnospiracaie, lactobacilli, and clostridial species. With the introduction of solids into the diet, bifidiobacteriae, enterobacterial, lactobacilli and clostridial species decline and the fiber fermenters lachnospiracaie, bacteriodes, and ruminococcace increase. There is also a fairly marked increase in the diversity of the fecal microbiome with the introduction of solids, and these changes appear independent of geographic location, mode of delivery, and whether the infant is breast and/or formula fed (52). Moreover, low microbial diversity early in life has been associated with an increased incidence of infantile colic, eczema, asthma and type 1 diabetes (53).

## Conclusions

There appears to be a sensitive period in the first several months of life when infants readily accept a wide variety of tastes and this period overlaps with a critical window for oral tolerance. As a result, it makes sense to expose infants to a wide variety of flavors while mother is pregnant, while mother is nursing and beginning at an early age. There also appears to be a sensitive period between 4 and 9 months when infants are most receptive to different food textures.

There remains debate as to when it is best to start introducing solid foods into an infant's diet however, the available evidence suggests provided the water and food supply are free of contamination, and the infant is provided adequate nutrition, there are no clear contraindications to feeding infants complementary foods at any age. There is emerging evidence that introduction of solid foods into an infant's diet by 4 months may increase their willingness to eat a variety of fruits and vegetables later in life, decrease their risk of having feeding problems later in life, and decrease their risk of developing food allergies, and the early introduction of solid foods into an infant's diet does not appear to increase their risk of obesity later in childhood.

## Author Contributions

SB is solely responsible for this manuscript. He conceived the paper, he solely wrote and edited the manuscript, and he has read and approved the final manuscript.

## Conflict of Interest

The author declares that the research was conducted in the absence of any commercial or financial relationships that could be construed as a potential conflict of interest.
